# Liposomes: The Brave Old World

**DOI:** 10.3390/ijms24054343

**Published:** 2023-02-22

**Authors:** Carla M. Matos

**Affiliations:** FP-I3ID (Instituto de Investigação, Inovação e Desenvolvimento Fernando Pessoa), FP-BHS (Biomedical and Health Sciences), Universidade Fernando Pessoa, Praça 9 de Abril, 349, 4249-004 Porto, Portugal; cmatos@ufp.edu.pt

Liposomes have been known of for about 60 years, since they were discovered by A. Bangham in the mid-1960s in a serendipitously brilliant finding [1]. The observation that adding a negative stain to dry phospholipids, when testing the institute’s new electron microscope, leads to the formation of a milky suspension, which was revealed to be composed of self-enclosed lipid bodies (liposomes). Immediately, their potential use in diverse research fields was perceived, and diverse areas of liposome use rapidly started to develop: as models for cellular membranes and drug-membrane interactions [2], as drug delivery and drug targeting tools [3], as microreactors for compartmentalizing reactional media [4], or in the development of chromatographic methods, used as the stationary phase [5]. One of the most interesting areas is using liposomes as site-specific drug delivery systems, improving the bioavailability and minimizing the systemic side effects; or for the topical administration of drugs, improving penetration through the skin [3]. A first drawback in the use of liposomes as drug delivery systems was their detection and capture by the body’s resticuloendothelial system (RES), namely the liver and the spleen. To avoid such a fate, PEG (polyethyleneglycol) is added to the outer layer as a way to disguise the lipid membrane (Stealth liposomes) and avoid RES. Other specific ligands can also be used to target specific delivery sites, as monoclonal antibodies (immunoliposomes) or antigens.

This Special Issue had the purpose to gather a collection of articles that highlight some recent liposome applications, such as the development of stealth liposomes to aim for the placenta, the use of liposomes as vaccine adjuvants, the development of prodrugs to increase liposome encapsulation and specific targeting, the development of theranostic (therapeutics and diagnostics) liposomes, and the use of elastic and ultradeformable liposomes for topical use.

One of the populations that can benefit from the use of drugs delivered in liposomes is pregnant women. However, to date, there are not enough studies on the pharmacokinetics and placental disposition of liposomes in this population. Fliedel et al. [6] presented a study with PEGylated liposomes and their uptake by the human placenta, using an in vitro model of human trophoblast cell line (BeWo cells) and an ex vivo model of suspended human villous placental explants for their study. Cationic liposomes exhibited a significant higher internalization compared to the neutral ones and exhibited an endocytosis mechanism of internalization via pathways implicating dynamin. These data highlight the key role of the liposome’s lipid composition and the possibility to modulate their internalization in the placenta by adjusting their design.

Zimmermann et al. [7] presented a work designed in the context of the recent SARS-CoV-2 pandemic, which was disruptive to the habits and planning of current societies. In the context of the development of new viral vaccines, against viruses such as SARS-CoV-1 and H5N1 influenza, among others, these authors suggest the use of liposomal vaccine adjuvant CAF^®^ 09b. This system induces a type I interferon response and can be used as a pan-viral prophylaxis prior to the development and approval of specific vaccines. Intranasal administration of these liposomes to mice showed an improved immunological defense response to the Influenza virus if administered prior to infection.

Kobanenko et al. [8] studied the drug colchicine (antimitotic and anti-inflammatory), frequently used as an acute gout flare treatment, in the form of a pro-drug (phospholipid-allocolchicinoid) as a way to incorporate the drug in the liposome bilayer and increase its encapsulation. As the complex does not have a pharmacological activity, but can be metabolized by hydrolysis to the active drug by means of the enzyme phospholipase A2, which exists in increased amounts in places where inflammatory processes are developing, this association constitutes an ingenious way of using the pro-drug concept to release the active ingredient colchicine in the site of action, increasing its selectivity and targeting. The use of liposomes further increases the passive targeting to inflammation or tumor sites.

Analytical techniques for the characterization of liposomes are also highlighted in this issue. Skupin-Mrugalska et al. [9] used AF4 (asymmetric field-flow fractionation) associated with MALLS (multi-angle laser light scattering) to elucidate the release mechanism of zinc phthalocyanine, a photosensitizing agent for photodynamic cancer therapy, from theranostic liposomes. In this study, liposomes had a dual function: first, as drug carriers, as the photosensitizing agent is usually a highly lipophilic compound, but that can easily incorporated in the liposome membrane; second, liposomes were used as an acceptor for the drug, to study the transference kinetics and the release mechanism to biological sinks, such as red blood cells.

Souto and co-workers [10] presented a review on the use of elastic and ultradeformable liposomes on transdermal delivery. The transdermal route is challenging, since the skin is a protection organ, built-up in order for protection and preventing the passage of exogenous molecules. The topical administration is intended to convey a local effect, and transdermal applications are employed to gain a systemic effect, avoiding the first-pass metabolism. Ultradeformable carriers (such as elastic liposomes) seem to be much more capable of permeating the skin, going deep enough to be absorbed by systemic circulation. The modification of the liposome composition can lead to a superior capability to enhance the transdermal drug delivery in comparison to conventional liposomes. Highly deformable liposomes, composed of a lipid mixed with some type of surface activator, were introduced, and are known as “elastic vesicles”. In that context, new vesicles, such as transfersomes, niosomes, ethosomes, and cerasomes, have been described.

## Data Availability

Not applicable.
